# Annual Meeting of the American Veterinary Medical Association

**Published:** 1901-06

**Authors:** 


### EDITORIALS.

### ANNUAL MEETING OF THE AMERICAN VETERINARY MEDICAL ASSOCIATION.

Every veterinarian in this country has a plain duty before him, and an obligation to perform to himself, to his family, to his clients, and to the profession by which he gains his living and makes his reputation. Each veterinarian should at once give his earnest consideration to the coming annual meeting of the American Veterinary Medical Association, which will take place at Atlantic City, N. J., on September 3d, 4th, and 5th, and learn what advantages this meeting will give him, and determine what he will do toward making the meeting a greater success than it would be without his presence.

No race-horse was ever kept in good form and fit to do its best, year after year, without an occasional rest, let-up in work, and change of diet. No veterinarian or other professional man can do proper, creditable, and his best work, either physically or mentally, without some relaxation at times, and change of thought and surroundings, to remove him from the narrowing rut, the wearing routine, and the jarring rust which will limit the energy and activity of any man, or irritate him into crotchets and vagaries.

The meetings of the A. V. M. A. have now become advanced scientific reunions. At them are to be heard the productions of the hardest working and most enthusiastic veterinarians in the country. The papers are almost all of determinate practical application. No matter how much a man reads in his office he fails there to get the true spirit of the discussion which is often the best part of a paper. Sitting in Q Street reading a book or the Journal, the opinions of Dr. Radical, from Boston; Dr. Quietman, from Helena; Dr. Practical, from a country town, or Dr. Bugs, connected with the laboratory of a hospital for two-legged patients, sound very differently from the same in words as heard at a meeting. Here the expression, the manner, the personality of the speaker tell you how accurate and honest he is, and forewarns you what confidence to place in his future views. A man's mind at a meeting is a camera from which he can draw living pictures to imitate or avoid at a future time in his own practice. The social intercourse at a meeting, the renewal of old friendships, the making of new acquaintances, the personal observation of others broadens a man's mind and cheers him up for return to the drudgery of his daily life.

Therefore, every veterinarian should arrange his summer so as to be present at the A. V. M. A., Atlantic City, September 3d, 4th, and 5th.

The wife has had her drudgery and routine through the year, and needs change. Atlantic City, the great seaside resort of America, offers the most attractive summer outing which can be made. The boardwalk, the piers, the music, the gowns, and hats of women from all parts of the country will compensate them for the care of the house at home for the rest of the year. In September it is still warm enough to bathe ; the surf on the beach and the roll on the ocean will repay any effort to make the visit from inland—New York or Philadelphia, with their shops and sights—for a day or two after the meeting adds to the attraction.

During September the hotel rates are considerably reduced from the rates of the arbitrary "season"—July and August. We believe some of the best hotels have given rates at $2 a day.

Members of the A. V. M. A. owe a duty to themselves and others to arrange to be present. All veterinarians, whether members or not, are cordially welcome to participate in the professional and social parts of the Association meetings. We are confident that they will find it will repay them to be present.

Let everyone begin his arrangements now to be at Atlantic City on September 3d, 4th, and 5th.

The July and August numbers of the JOURNAL will give full details as to programme, transportation, hotel accommodations, etc.

